# Attenuation of Hypothyroidism-Induced Cognitive Impairment by Modulating Serotonin Mediation

**DOI:** 10.3390/vetsci10020122

**Published:** 2023-02-06

**Authors:** Dimitar Bakalov, Petar Iliev, Zafer Sabit, Radka Tafradjiiska-Hadjiolova, Georgeta Bocheva

**Affiliations:** 1 Department of Physiology and Pathophysiology, Medical University of Sofia, 1431 Sofia, Bulgaria; 2 Department of Pharmacology and Toxicology, Medical University of Sofia, 1431 Sofia, Bulgaria

**Keywords:** hypothyroidism, cognitive impairment, depressive-like behavior, tryptophan

## Abstract

**Simple Summary:**

Propylthiouracil (PTU) is commonly used to create a model of hypothyroidism, one of the most common diseases worldwide that has a significant social impact. This study aimed to examine the changes in the behavior, cognition, and memory in rats with PTU-induced overt hypothyroidism as well as the effects of tryptophan treatment on this state. For this purpose, we administered 5-OH-tryptophan intraperitoneally or directly into the hippocampus of the hypothyroid animals. Their behavior and cognition were assessed using an open field test, T-maze, and novel object recognition test. There were significant differences in the behavioral patterns of the hypothyroid animals, showing a reduction in locomotor activity, rearing, and memory function compared to the controls. The treatment with 5- hydroxy-tryptophan (5-OH-TRP) alleviated those changes. A staggering amount of research is suggesting that the usual denominators in the pathophysiology of depression and the cognitive decline in hypothyroidism are the hippocampal complex and the distorted serotonin metabolism. In our study, we observed significant beneficial effects on cognitive impairment after 5-OH-TRP administration. Current results are promising and may serve as groundwork for further investigation of functional and structural changes in the hippocampus during a hypothyroid state, and in particular, the effects of serotonin mediation in hypothyroid-associated depressive behavior.

**Abstract:**

Thyroid hormones play an important role in the modeling of neural networks in the brain. Besides its metabolic effects, thyroid dysfunction, and hypothyroidism in particular, is frequently associated with cognitive decline and depressive-like behavior. The current study aimed to examine the changes in behavior, cognition, and memory in rats with propylthiouracil-induced overt hypothyroidism. The behavior and cognition were assessed using the open field test, T-maze, and novel object recognition test. We found significant differences in the behavioral patterns of the hypothyroid animals showing a reduction in locomotor activity, frequency of rearing, and impaired memory function compared to the euthyroid controls. As serotonin is an essential biomarker regulating cognition and mood, we tried to modulate the serotonin mediation in hypothyroid animals through tryptophan administration. Treatment with 5-hydroxy-tryptophan (5-OH-TRP) intraperitoneally for 10 days or directly into the hippocampus as a single injection led to attenuation of the hypothyroidism-induced cognitive and memory decline. A staggering amount of research is suggesting that the common denominators in the pathophysiology of depression and the behavior changes in hypothyroidism are the hippocampal complex and the distorted serotonin metabolism. In our study, it was observed a significant alleviation of cognitive impairment and an improvement of memory performance in hypothyroid rats after 5-OH-TRP administration. Current results are promising and may serve as groundwork for further investigation of functional and structural changes in the hippocampus during a hypothyroid state, and in particular, the effects of serotonin mediation in hypothyroid-associated depressive-like behavior.

## 1. Introduction

Hypothyroidism is one of the most common metabolic disorders with varying distribution throughout the world according to local dietary specifics and demographic characteristics. Because of the extensive role of thyroid hormones in the regulation of the metabolism, the symptoms of hypothyroidism are frequently attributed to other diseases [1,2]. Large observational studies and meta-analyses have shown that about 4–7% of community-derived populations in the USA and Europe have undiagnosed hypothyroidism. Nearly 80% of the cases have subclinical hypothyroidism with the remainder having overt hypothyroidism [3]. Notably, thyroid hormones play an important role in the regulation of brain development in fetuses and neonates, but also in maintaining synaptic plasticity, learning, and memory processes in adults [4,5]. The role of the thyroid gland in the proper function of the adult brain becomes even more evident with significant epidemiological data showing that hypothyroidism frequently coexists with depression or dementia and the positive effect of thyroid hormone supplementation in patients with drug-resistant depression [6,7]. A large-scale study showed that individuals with hypothyroidism faced a higher risk of memory impairment (up to 81%), higher perceived stress, more than a 3-fold increase in dementia risk, higher rates of depression and anxiety, greater fatigue, poorer concentration, and less motivation [8]. Some rare cases of autoimmune thyroiditis could lead to Hashimoto’s encephalopathy, presented by nonspecific clinical manifestations rapidly leading to progressive dementia, reversed by corticosteroid therapy [9,10].

In our study, we focused on another essential biomarker regulating cognition and mood: serotonin (5-HT), implicated in the pathogenesis of hypothyroidism-associated cognitive impairment [11,12,13]. Increased brain 5-HT concentration has been proven to enhance cognitive function [14], whereas decreased 5-HT metabolism in the brain has been shown to impair memory and cause depressive-like behavior [15,16]. Tryptophan (TRP), an essential amino acid, is the sole precursor of 5-HT. The serotonergic system has a significant role in memory regulation in the hippocampus [14,17]. Many brain regions are involved in the learning process, but the hippocampus has a key role in learning and memory. TRP depletion is well known for its detrimental effects on this system, such as disturbed novel object recognition [18].

Chemically induced hypothyroidism is a well-established model in the literature for studying the depressive-like behavior often found in this state [19,20]. The aim of the current study was to examine the behavioral changes and cognitive performance in a rat model of propylthiouracil-induced overt hypothyroidism and the effects of TRP, administrated either intraperitoneally (i.p.) or directly in the hippocampus. TRP administration enhances its availability in the brain and thus potentially increases 5-HT synthesis. We studied the behavior, cognition, and memory characteristics of hypothyroid animals untreated and treated with TRP 

## 2. Methods

### 2.1. Ethics Statement

All animals were treated in agreement with the general regulations for the treatment of experimental animals, established by the Ethics Committee of the Medical University of Sofia and the Bulgarian Agency for Food Safety (approval No. 319/2021 from BAFS), in agreement with EU Directive 2010/63/EU for the protection of animals used for scientific purposes.

### 2.2. Drug-Induced Hypothyroidism

Hypothyroidism was induced in 20 male Wistar albino rats with an average starting body weight of about 210 g by administration of 0.01% 6-*n*-propyl-2-thiouracil (PTU) (Sigma-Aldrich^®^, St. Louis, MO, USA) for 5 weeks in the ad libitum consumed drinking water [1,21,22]. After reaching the hypothyroid state, proofed by free T4 (fT4) levels in the serum (measured by chemiluminescent immunoassay), 5 animals were injected intraperitoneally (i.p.) with 50 mg/kg [23,24] of 5-hydroxy-L-tryptophan (5-OH-TRP) (Sigma-Aldrich^®^, St. Louis, MO, USA) in 1% PBS, 5 animals were subjected to stereotactic hippocampal injections of 3 µL solution of 5-OH-TRP (17 mg/mL), and 10 hypothyroid rats were treated with only 1% PBS (either i.p. or by stereotactic injection) for 10 days.

### 2.3. Open Field Test (OFT)

The device consists of a box with dimensions of 60 × 60 × 35 cm, divided into two zones—external (60 × 60 cm) and internal (35 × 35 cm) squares and a video camera connected to software recording the movement of the experimental animal (OBS Studio, https://obsproject.com/). The procedure starts with placing the test animal in the center of the box and tracking the length of the trajectory and the time the animal stays in the inner and outer square over 10 min. The video analysis is performed with specialized ToxTrack software (version 2.93) [25]. The inner zone is perceived as aversive and the stay duration and the length of the trajectory traveled in it, calculated as a percentage of the total value, express the level of anxiety. The total number of entries into the central area is considered an indicator of research behavior, together with horizontal activity and rearing.

### 2.4. T-Maze with Spontaneous Alternation

The device (T-maze) is constructed as an initial alley (15 cm × 35 cm) connected perpendicularly in the middle of an alley measuring 12 cm × 85 cm, forming a T-shape with two arms 12 cm × 35 cm. A plexiglass barrier was placed at both entrances to the shoulders. The test was performed according to Gerlai’s procedure (1998). The rat was first directed to the left shoulder by blocking the entrance to the right one with a plexiglass barrier. When the rat returned to the starting alley after examining the left shoulder, the barrier was removed. The rat was allowed to descend the alley and choose one of the two shoulders. Once the rat selected one arm, the opposite was blocked by the barrier. The free selections were monitored continuously for 10 min which was the total time for research and percentage of alternation (alternations must be above 50% to exclude change of direction due to chance).

### 2.5. Novel Object Recognition Test (NOR)

The device is an opaque box (60 × 60 × 35 cm), located in a soundproofed, evenly-lit room. The procedure consists of 3 phases: on the first day—getting used to the new environment for a period of 15 min; on the second day—5 min of training by using two identical objects placed in the box, and the time during which the animal examines them is recorded; phase 3—a 5 min test one hour after the training session. During this phase, one of the already studied objects is replaced with a new one which has a different shape and color. The time for investigation of the familiar and the novel object is taken into account. The objects are selected in order not to resemble food and water and not to have a specific smell. Research behavior is defined as directing the animal’s nose to the object at a distance of fewer than two centimeters, accompanied by a vibration of the whiskers. The ability to distinguish the new from the already known object is presented as the recognition index (RI).

$$I=\frac{time\;of\;research\;of\;the\;new\;object\;\times \;100\%}{time\;of\;research\;of\;the\;new\;object\;+\;time\;of\;research\;of\;the\;known\;object}$$

After each of the behavioral tests, the apparatus is thoroughly cleaned with 0.1% acetic acid solution to remove olfactory traces. 

### 2.6. Stereotactic Injection

**Figure 1:**
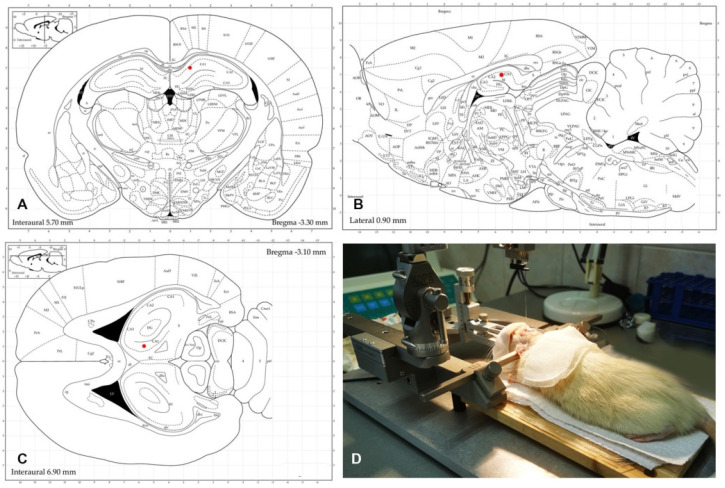
Stereotactic injection of 5-OH-TRP according to the Paxinos and Watson (1998) stereotactic coordinates of the hippocampal complex (marked as red dots). (**A**–**C**) Coronal, sagittal, and axial plains. (**D**) One of our experimental animals during the surgery.

After anesthesia with ketamine and xylazine, an incision was made in the skin of each animal to reveal the skull, and after a careful cleaning of the aponeurosis, the lambda and bregma were detected. Subsequently, and in accordance with stereotactic coordinates ML = 1 mm, AP= −3.4 mm, and DV = 3 mm (Figure 1A,B,C - according to the Atlas of Paxinos and Watson, 1998), the hippocampus was reached, where 3 µL solution of 5-OH-TRP (17 mg/mL) or 1% PBS at a rate of 1 µL/min were injected using a Hamilton syringe (Figure 1D).

This was followed by the closure of the operative field and follow-up of the animal in a separate clean cage. The animal was subjected to behavioral tests 7 days after the stereotactic injection.

### 2.7. Statistics

All data from behavioral experiments are presented as means ± SD. Statistical analysis was performed with two-way ANOVA with the following factors: thyroid status (control euthyroid levels and hypothyroidism) and treatment (saline and 5-OH-TRP) with Bonferroni post-test. *p* < 0.05 is considered statistically significant. Software used was Sigma Plot 11.0 and GraphPad Prism 9.0.

## 3. Results

### 3.1. fT4

A significant difference (*p* < 0.001) was observed in the PTU-treated group (0.64 ± 0.36 ng/dl) compared to the control euthyroid < 0.001) in the serum concentration of fT4 levels (*p* < 0.001) was observed in the PTU-treated group (0.64 ± 0.36 ng/dl) compared to the control euthyroid group (20.40 ± 3.99 ng/dl), confirming the induction of a hypothyroid state.

### 3.2. Open Field Test

**Figure 2:**
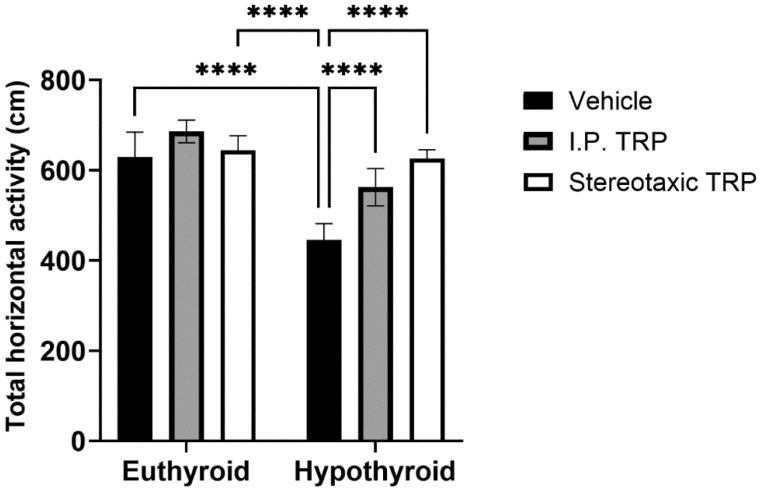
Effects of hypothyroidism and 5-OH-TRP treatment in both hypothyroid and euthyroid rats on the total horizontal activity (OFT) in male Wistar albino rats; ****—*p* < 0.0001.

The experimental data showed that PTU-induced hypothyroidism significantly changed the total horizontal motor activity (F (1, 24) = 76.84, *p* < 0.05). The i.p. administration of 5-OH-TRP for 10 days and the direct single stereotactic injection in hypothyroid rats normalized their motor activity (F (2, 24) = 25.38, *p* < 0.05; Figure 2) without a significant difference between the two routes of treatment (*p* = 0.084).

**Figure 3:**
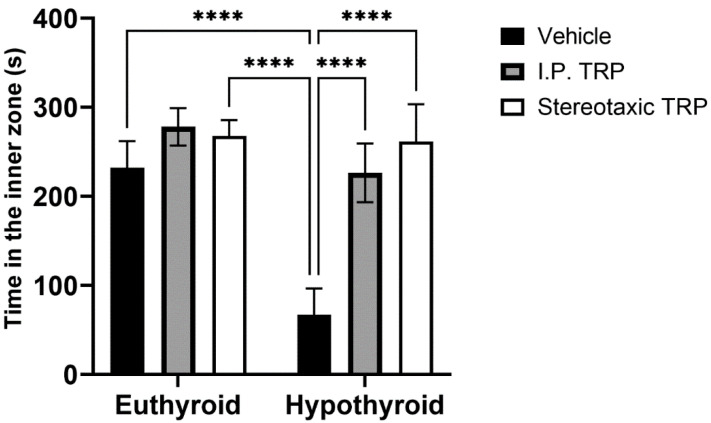
Effects of hypothyroidism and 5-OH-TRP treatment in both hypothyroid and euthyroid rats on the exploratory behavior (OFT) in male Wistar albino rats; ****—*p* < 0.0001.

**Figure 4:**
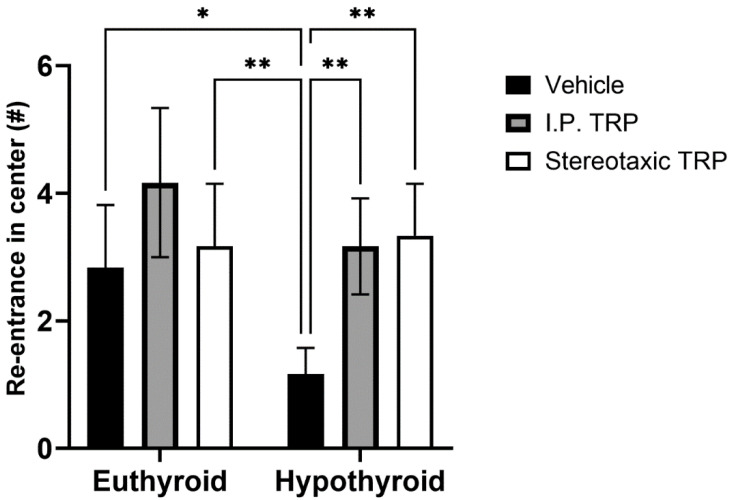
Effects of hypothyroidism and 5-OH-TRP treatment in both hypothyroid and euthyroid rats on the time spent in the inner zone during OFT; *—*p* = 0.030; **—*p* = 0.0059.

Hypothyroidism intensified the anxiety-like behavior by significantly reducing the length of the trajectory in the central aversive part of the apparatus (two-way ANOVA F (1, 24) = 55.82; *p* < 0.05), while i.p. treatment and the direct stereotactic injection with 5-OH-TRP significantly and comparably increased the distance traveled in the center (F (2, 24) = 53.66; *p* < 0.05) (Figure 3) and increased the number of re-entries in the center of the device (F (2, 24) = 11.52; *p* = 0.0002), causing a pronounced anxiolytic effect (Figure 4).

**Figure 5:**
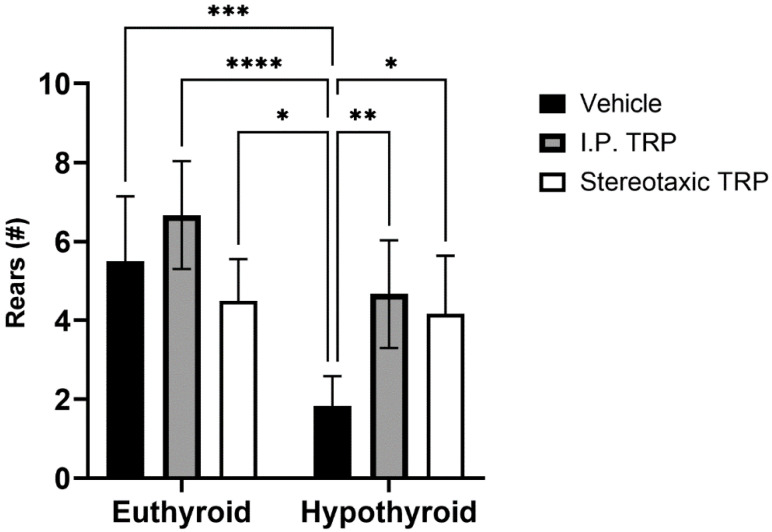
Effects of hypothyroidism and 5-OH-TRP treatment in both hypothyroid and euthyroid rats on the exploratory behavior (OFT) in male Wistar albino rats; *—*p* = 0.03; **—*p* = 0.0058; ***—*p* < 0.005; ****—*p* < 0.001.

Furthermore, our tests showed that hypothyroidism caused suppressed exploratory behavior by significantly reducing the number of times the animals reared (two-way ANOVA F (1, 16) = 27.52; *p* < 0.05), while 5-OH-TRP i.p. treatment for 10 days significantly increased the number of times the hypothyroid animals reared (F (1, 24 = 12.53; *p* < 0.05) (Figure 5) suppressing the effects of hypothyroidism. It was observed that hypothyroid animals treated with a stereotactic injection of 3 µL solution of 5-OH-TRP (17 mg/mL) in the hippocampal formation demonstrated a significant improvement in the behavioral markers, being similar to the results from rats treated with 5-OH-TRP i.p. for 10 days.

### 3.3. Novel Object Recognition Test

**Figure 6:**
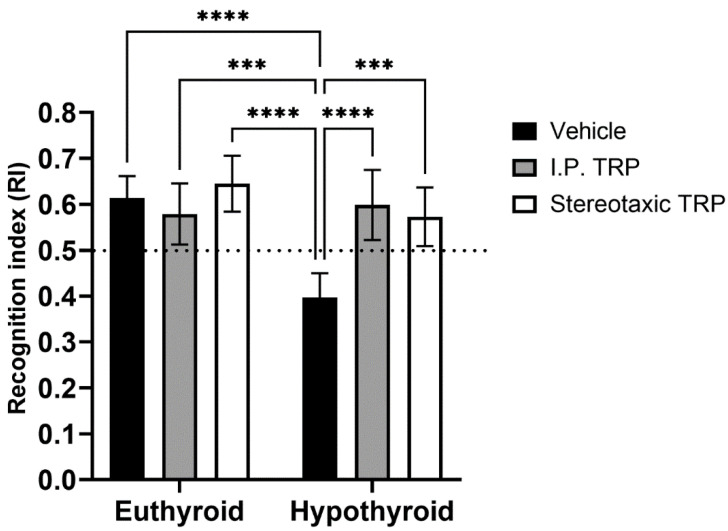
Influence of PTU-induced hypothyroidism and 5-OH-TRP treatment on the working memory in the novel object recognition test of male Wistar albino rats. *** *p* < 0.01, **** *p* < 0.001.

**Figure 7:**
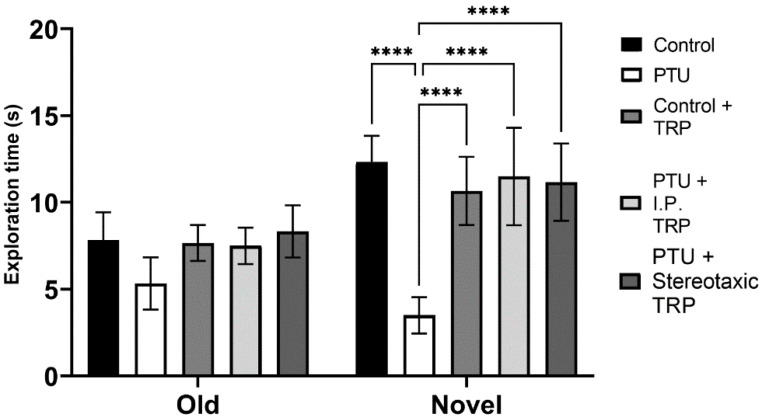
Influence of hypothyroidism and 5-OH-TRP treatment on the exploration time in the novel object recognition test of male Wistar albino rats. Data are presented as means ± SD, *p* < 0.05 compared to 5-OH-TRP-treated hypothyroid rats. **** *p* < 0.005.

The RI of the new object from the already known one showed that hypothyroidism impaired working memory (F (1, 24) = 18.89, *p* = 0.0001), whereas TRP administration (either i.p. or stereotactic) relieved amnesia caused by hypothyroidism to levels comparable to euthyroid controls (F (2, 24) = 9.397, *p* = 0.007) (Figure 6). Here, as in OFT, it was observed that untreated hypothyroid animals showed significantly lower exploratory behavior (F (4, 40) = 22.63; *p* < 0.05) (Figure 7).

### 3.4. T-Maze Test

To study their working memory, the animals were tested for their spontaneous alternation in the T-maze. The difference in the alternation percentage (mean ± SD) of the hypothyroid animals (44.00 ± 5.477) was statistically significant (F (1, 24) = 13.66; *p* = 0.0001) compared to 78.00 ± 8.367 for euthyroid controls, 73.69 ± 4.159 for euthyroid controls treated i.p. with 5-OH-TRP, 79.09 ± 7.416 for euthyroid controls treated stereotactically with 5-OH-TRP, 68.00 ± 4.472 for hypothyroid animals treated i.p. with 5-OH-TRP, and 68.00 ± 7.583 for hypothyroid animals treated stereotactically with 5-OH-TRP.

## 4. Discussion

For our cognitive tests, Wistar albino rats were used to study hypothyroid-associated behavior changes. We have previously published that PTU-induced hypothyroid rats demonstrated a depressive-like behavior tested by the forced swimming test (FST) [21]. Additionally, we have already reported that hypothyroidism significantly alters the levels of 5-HT in the brains of hypothyroid compared to euthyroid animals [22].

Although brain thyroid hormone levels do not correlate with their concentration in the blood, there are reports that the thyroid hormone content in the hippocampus is decreased in Wistar albino and Wistar–Kyoto (WKY) rats receiving PTU [13].

In OFT, we observed that PTU-induced hypothyroidism significantly decreased total horizontal locomotor activity, which confirms several previous reports [26,27,28]. There was also a diminished risk-taking behavior, demonstrated by reducing the number of re-entries to the central aversive area of the OFT apparatus. The decrease in locomotor behavior could be explained by either hypoactivity or by a reduction in exploration. The activity pattern is less vulnerable in the adult period, and the decrease in observed locomotion may be a result of reduced exploration in the hypothyroid rats. The treatment with 5-OH-TRP almost completely abolished hypothyroid-induced hypoactivity and reduced risk-taking behavior in experimental animals, but with a slight difference; it was not significant compared to the baseline, which demonstrated a marked anxiolytic effect from the administrated 5-OH-TRP (i.p. or stereotactic). At the same time, although tryptophan treatment of euthyroid animals demonstrated a tendency to increase locomotor activity, compared to the untreated controls, it was not statistically significant.

Another important marker of exploration in OFT is rearing, which is one of the most common responses of an animal exposed to an unknown environment. In the current study, we observed significantly reduced rearing in PTU-induced hypothyroid rats, which was reflected by their low exploratory motivation. Sapronov et al. described this phenomenon of decreased rearing in adult rats after thyroidectomy; however, it was found to be not significant [29]. On the other hand, several studies in adult rats with PTU-induced hypothyroidism have found significant impairments in spatial learning and memory [30,31] and changes in the CA1 area of the hippocampus. These changes include alterations in the expression of a gene called neurogranin (RC3), a gene regulated by the thyroid hormones, which is involved in memory formation and synaptic plasticity [32]. Treatment with PTU has been found to reduce brain and hippocampus volume and alter the expression of RC3, calmodulins (CaMs), and ERK, important for the animals’ spatial memory [33]. Notably, it was reported that PTU stimulates the development of neuroinflammation, Aβ production, tau hyperphosphorylation, and altered neuroplasticity of the hippocampus leading to memory deficits [33,34]. The exploratory behavior is frequently diminished in affective disorders, and rats subjected to chronic stress, as a model of depression, have been found to display a significantly reduced rearing in OFT [35]. Furthermore, because exploration is rewarding, this was speculated to be a sign of anhedonia, a hallmark of depression [36].

A similar pattern was observed in the NOR test. The total time spent exploring the novel object in the NOR test session by the hypothyroid rats was significantly decreased compared to that spent exploring the familiar object, and the total exploration time in hypothyroid rats was delayed in comparison with the euthyroid controls, demonstrating a reduction in short-term memory. On the other hand, there was no significant difference in the time spent exploring the objects by 5-OH-TRP-treated euthyroid rats compared to the untreated control group. These results indicated that the memory formed in the familiarization session was retained for at least 1 h in the group that received 5-OH-TRP. The NOR task evaluates the rodents’ ability to recognize a novel object in the environment. The dorsal hippocampus plays an important role in memory formation, especially when spatial or contextual information is a relevant factor, as in the NOR test [37,38].

**Figure 8:**
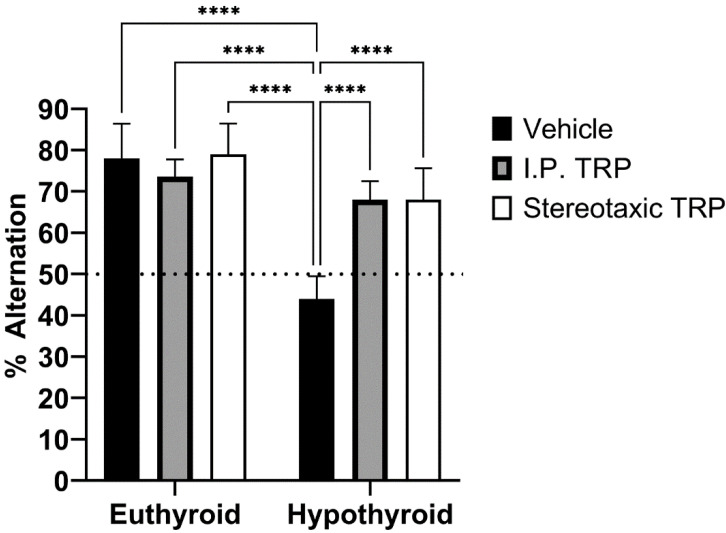
Influence of hypothyroidism and 5-OH-TRP treatment on the alternation percentage in the T-maze test; **** *p* < 0.0001; ns—non-significant.

At the same time, untreated euthyroid rats and 5-OH-TRP-treated hypothyroid animals demonstrated similar choice accuracy during the acquisition of T-maze alternation behavior, showing a positive effect of TRP supplementation—either i.p. or intracerebral. Taken together, these data indicate that rats in a hypothyroid state have a deficit in the retention of spatial information for a longer period, which is a process normally dependent on the hippocampus. Data presented in Figure 8 indicate that 5-OH-TRP administration increased the number of alternations during acquisition and did affect the rate of acquisition by diminishing the amnesic effects of hypothyroidism.

The modulation of tryptophan and 5-HT by thyroid hormones has been well documented in the brain areas of developing hypothyroid rats [39,40,41], but the effect of hypothyroidism on 5-OH-TRP metabolism and turnover of catecholamines is still not fully understood in adult rats. Based on previously published data on hyperthyroidism [42] and hypothyroidism [43], we could speculate that in a hypothyroid state, the level of 5-OH-TRP, the activity of tryptophan hydroxylase, and the synthesis of 5-HT might be reduced in some areas of the brain, such as the hypothalamus.

Our results confirm previous reports that serotonin mediation can counteract the effects of hippocampal dysfunction caused by hypothyroidism [29,44]. Thus, the behavioral, learning, and memory effects of TRP may be due to its beneficial influence on the hippocampus. This assumption is supported by increasing evidence showing that the neurotransmitter serotonin is a key regulator of the hippocampal functions, being directly involved with the active learning process and memory [45,46,47]. Both serotonin depletion and specific serotonin antagonists can lower memory performance. However, serotonin does not cross the blood–brain barrier, and its synthesis depends on the intake of amino acids, such as tryptophan [48].

Studies evaluating the effects of increased hippocampal serotonin metabolism following 5-OH-TRP administration showed enhanced short- and long-term memory, improved learning acquisition, and memory consolidation [49].

## 5. Conclusions

A staggering amount of research is suggesting that the usual denominators in the pathophysiology of depression and the cognitive decline in hypothyroidism are the hippocampal complex and the distorted serotonin metabolism. In our study, we explored the behavioral and memory effects of overt hypothyroidism and observed significant beneficial effects on cognitive impairment after 5-OH-TRP administration. Current results are promising and may serve as groundwork for further investigation of functional and structural changes in the hippocampus during a hypothyroid state, and in particular, the effects of serotonin mediation in hypothyroid-associated depressive behavior.

## Data Availability

All data appear in the manuscript. For further inquiries, please contact the first author or corresponding author.
